# Clinical course, viral etiology, and the diagnostic workup for patients with suspected myocarditis: a single-center prospective study

**DOI:** 10.1186/s12872-022-02833-0

**Published:** 2022-09-06

**Authors:** Shimaa S. Khidr, Mohamed Ahmed El-Mokhtar, Shery Refaat Asaad, Helal F. Hetta, Mona Hussein Abdel-Rahim, Amr Ahmed Aly Youssef, Ayman K. M. Hassan

**Affiliations:** 1 Department of Cardiovascular Medicine, Faculty of Medicine, Assiut University, P.Box. 71526, Assiut, Egypt; 2 Department of Microbiology and Immunology, Faculty of Medicine, Assiut University, Assiut, Egypt

**Keywords:** Myocarditis, Clinically suspected myocarditis, Cardiac magnetic resonance imaging, Cardiotropic virus, Polymerase chain reaction, ELISA, Endomyocardial biopsy

## Abstract

**Background:**

Myocarditis is a highly heterogeneous disorder with a challenging diagnostic work-up. We aimed to focus on the possible diagnostic workup for this condition in settings where endomyocardial biopsy as a gold standard is not always feasible, detect the etiologic cardiotropic viruses in our locality, and follow the clinical course in patients admitted with clinically suspected myocarditis.

**Methods:**

This is a prospective observational study. We recruited patients with clinically suspected myocarditis presenting at a university hospital from October 1st, 2020 until March 31st, 2021. All Patients had a diagnostic coronary angiography and were included only if they had a non-obstructive coronary artery disease. All patients also had cardiac magnetic resonance imaging (CMR) with contrast. Sera were obtained from all suspected patients for detection of antibodies against viruses using enzyme-linked immunosorbent assay, and viral genomes using polymerase chain reaction (PCR), and reverse transcription–PCR. Endomyocardial biopsy was done for patients with a typical CMR picture of myocarditis.

**Results:**

Out of 2163 patients presenting to the hospital within the 6 months, only 51 met the inclusion criteria. Males represented 73%, with a mean age of 39 ± 16 years. CMR showed an ischemic pattern in 4 patients and thus they were excluded. We classified patients into two categories based on CMR results: group A (CMR-positive myocarditis), 12 patients (25.5%), and group B (CMR-negative myocarditis), 35 (74.5%) patients. On serological analysis, 66% of patients (n = 31/47) showed antibodies against the common cardiotropic viruses. Parvovirus B19 IgM in 22 patients (47%) and coxsackievirus IgM in 16 (34%) were the most observed etiologies. Regarding the outcome, 42.5% of patients recovered left ventricular ejection fraction and three patients died at 6 months’ clinical follow-up.

**Conclusion:**

Patients with Clinically suspected myocarditis represented 2.2% of total hospital admissions in 6 months. CMR is only a good positive test for the diagnosis of acute myocarditis. Parvovirus B19 and coxsackievirus were the most common pathogens in our locality.

*Trial registration*: Clinical trial registration no., NCT04312490; first registration: 18/03/2020. First recruited case 01/10/2020. URL: https://register.clinicaltrials.gov/prs/app/action/SelectProtocol?sid=S0009O3D&selectaction=Edit&uid=U0002DVP&ts=2&cx=9zdfin.

## Introduction

Viral myocarditis seems to be the most common infectious etiology of acute myocarditis worldwide [1–3]. The incidence of acute myocarditis is estimated to be 1.5 million cases per year globally. The contribution of myocarditis as a cause of heart failure varies by age and region from approximately 0.5–4.0% [3, 4]. The clinical manifestations of viral myocarditis are highly variable, and the diagnostic workup of myocarditis is a dilemma. According to the European Society of Cardiology guideline scoring myocarditis [5], clinically suspected myocarditis is defined in the presence of ≥ 1 clinical presentation and ≥ 1 diagnostic criteria from different categories in the absence of angiographically detectable coronary artery disease (CAD) [5–7]. Cardiac magnetic resonance imaging (CMR) is a non-invasive tool commonly used for the diagnosis of acute myocarditis, which is sensitive to the tissue changes that occur during myocardial inflammation. However, its limited time window for detection (in 1st 10–15 days of presentation) and different software programs used, resulted in a reduced negative predictive value of CMR in acute myocarditis [8]. Endomyocardial biopsy (EMB) is considered the most accurate diagnostic modality for myocarditis [9, 10]. However, due to its invasive nature, its indications vary among societies [3, 11, 12]. Serological tests include enzyme-linked immunosorbent assay [13] and polymerase chain reaction (PCR) are sometimes used in the diagnostic workup to can help detect the causative virus [14, 15]. However, in some cases still, most of these techniques may reveal negative results and the suspected diagnosis might not be confirmed. Thus, in clinical practice, we still don’t have a simple gold standard to diagnose these patients.

The prevalence of specific cardiotropic viruses is variable over time, Parvovirus B19 (PVB19) and human herpes virus-6 (HHV6) have been increasingly detected in EMB of patients with acute myocarditis over the past 20 years [16]. A recent report from South Africa showed an almost similar distribution [16]. It is not clear yet if this is the case in Egypt or not.

Since there is limited data regarding the prevalence of viral pathogens associated with myocarditis in Egypt, this study aimed to examine the percent of clinically suspected myocarditis within hospital admitted patients, detect their different clinical presentations and outcomes, focus on the feasible diagnostic workup techniques, and identify the prevalent etiologic cardiotropic viruses in our locality.

## Methods

### Study group and design

**Fig. 1:**
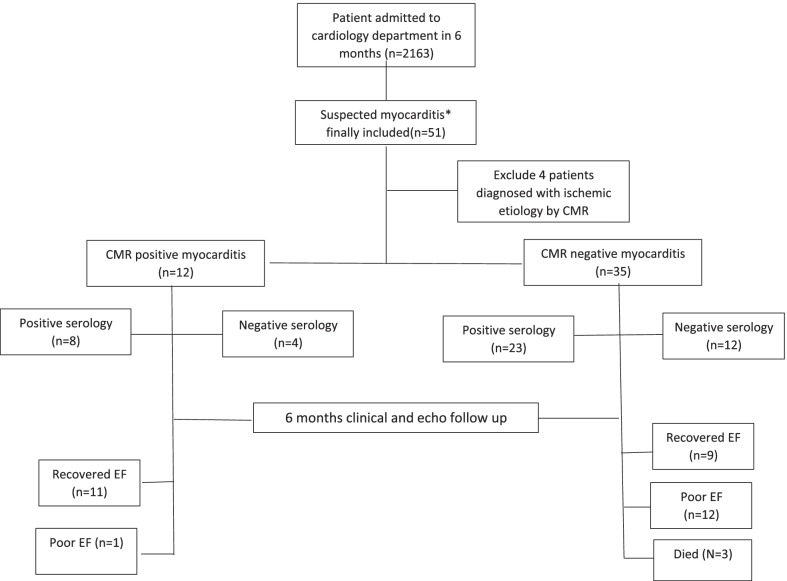
Flow chart of the study groups with clinical follow-up at 6 months

This was a prospective observational study. Patients were recruited between October 2020 and March 2021. A total of 2163 patients were admitted to the cardiovascular medicine department at a University Heart Hospital during this period. Our inclusion criteria were based on the ESC guideline scoring for suspected myocarditis [5]. All included patients had a score ≥ 2. Fifty-one patients with suspected myocarditis fulfilled the inclusion criteria (Fig. 1).

We excluded patients with known or recently diagnosed ischemic heart disease or significant coronary artery disease (on coronary angiography), valvular, congenital, or infiltrative heart disease (confirmed on echocardiography or the CMR study). We also excluded patients with uncontrolled hypertension, diabetes, peripartum cardiomyopathy, cardiotoxic exposure, alcoholic patients, those with a history suggestive of familial cardiomyopathies, and critically ill patients, unable to undergo CA or CMR.

According to the ESC position statement [15], clinically suspected myocarditis is defined in presence of ≥ 1 clinical presentation and ≥ 1 diagnostic criteria from different categories in the absence of angiographically detectable coronary artery disease (coronary stenosis, ≥ 50%) and known preexisting cardiovascular disease or extracardiac causes which could explain the syndrome (e.g., valve disease, congenital heart disease, hyperthyroidism). Suspicion is higher with a higher number of fulfilled criteria; if the patient is asymptomatic, ≥ 2 diagnostic criteria should be met. Clinical presentation could include acute chest pain, new-onset dyspnea (days up to 3 months), subacute/chronic dyspnea [> 3 months], (in our study, only patients with symptom onset < 3 months duration were included), palpitations and/or unexplained arrhythmia and\or unexplained cardiogenic shock. Diagnostic criteria are defined as follows: ECG features of cardiac damage, elevated markers of myocardial necrosis, functional and/or structural abnormalities on cardiac imaging (echocardiogram or angiogram or CMR), and tissue characterization by CMRI (edema and/or LGE of classical myocarditis pattern).

The trial protocol was reviewed and approved by the institutional review committee (IRB no = 17400018). All patients provided written informed consent to participate in the trial. The demographic and clinical data were collected using a standardized “procedural datasheet” (clinical trial registration no., NCT04312490; STDF grant no., 26393).

### Patients’ diagnosis protocol

All patients were subjected to the following during their course of admission.

#### Full history-taking

Full history-taking included history and current complaint, stressing the most common symptoms of myocarditis, such as chest pain, shortness of breath, fatigue, palpitations, and fainting attacks; history of flu-like symptoms or a preceding viral infection (cough, fever, malaise); history of previous COVID 19 infection and history of any systemic disease, toxic agents (chemotherapy, alcohols, drugs), family history of cardiac or neuromuscular disease or sudden cardiac death in family members at a young age (< 50 years).

#### Full physical examination

Full physical examination including cardiac examination.

#### Twelve-lead ECG

This was conducted with special attention to ECG findings suggestive of myocarditis as tachycardia, PR prolongation, ST-segment deviation, and poor R wave progression.

#### Echocardiography

All enrolled patients underwent transthoracic echocardiography, which was conducted within 24 h from admission by the same operator and same machine (General Electric Vivid 5) using an S3 probe for 2D data. Segmental wall motion and pericardial abnormalities were determined by 2D imaging. Valve lesions were examined using color Doppler, continuous-wave Doppler, and pulsed-wave Doppler. Left ventricular [4] ejection fraction (EF) and cardiac dimensions were determined using standard protocols by experienced physicians.

#### Cardiac catheterization

All cases were examined on the day of admission at the university heart hospital catheterization laboratory (Philips Allura Xper Fd 10). In all cases, the femoral artery was cannulated with a 6-Fr sheath, and coronary artery cannulation was performed using a 6-Fr guiding catheter. All cases were examined by a professional interventional cardiologist in a timely manner according to guidelines.

#### CMR

Performed within 1–2 days of admission using a 1.5-Tesla MRI scanner (Philips Ingenia Release 4.1.3.0, Philips Medical Systems, the Netherlands), using a phased array cardiac receiver coil.

All patients underwent standard cine steady-state-free precession images of the left and right ventricles in the horizontal and vertical long-axis views and left ventricular outflow tract view, and a stack of short-axis images for volumetric and functional assessment of the left and right ventricles (TR/TE: 3.1 ms/1.5 ms, flip angle 70°, FOV: 300 mm, Voxel size: 1.97/2.05/8.00 mm, 8 mm slice thickness with no gaps for short-axis images). Then, T1 (TR/TE: 674.2 ms/4.0 ms, flip angle 90°, FOV: 300 mm, Voxel size: 1.39/1.8/8.00 mm), and edema-sensitive black-blood T2-weighted STIR sequences (TR/TE: 1348.3 ms/70 ms, flip angle 90°, FOV: 300 mm, Voxel size: 1.5/2.14/8.00 mm) were taken, in basal, mid and apical levels of the short axis view of both RV and LV, and in long-axis views (2–3 and 4 chamber views), to be able to correlate any changes detected in 2 perpendicular views.

For contrast enhancement, a bolus of 0.2 mmol/kg of body weight of Gadodiamide (gadolinium-based contrast agent) was administered intravenously, LGE images were acquired between 10 and 20 min, by phase-sensitive inversion recovery technique, in 2, 3, and 4 axis views, together with 3–5 short-axis levels.

##### Image analysis

CMR analysis was performed offline by using dedicated software (MR Workspace R 2.6.3.1).

Diagnosis of myocarditis was based on the modified Lake Louis criteria using the regional or global increase in T2 signal intensity and area of increased signal intensity in a non-ischemic distribution in late gadolinium enhancement (LGE) images (parametric mapping is not available yet in our center). Cases were reported positive when they had both T2 and LGE features. Other supporting features were also sought, such as pericardial effusion, increased pericardial signal intensity in T2 or LGE sequences, and regional or global wall-motion abnormalities in cine sequences [17].

In post-processing of the cine images, endocardial and epicardial contours were traced on end-diastolic and end-systolic frames to calculate LV and RV end-diastolic volumes, end-systolic volumes, mass, ejection fraction, and  stroke volume, all indexed to body surface area. SSFP sequences were also examined for any myocardial or pericardial thickening, segmental wall motion abnormalities, pericardial effusion, and any other cardiac or extracardiac abnormalities.

The presence of focal regional high signal intensities on T2 STIR and LGE images in a nonischemic distribution pattern was assessed visually.

**Fig. 2:**
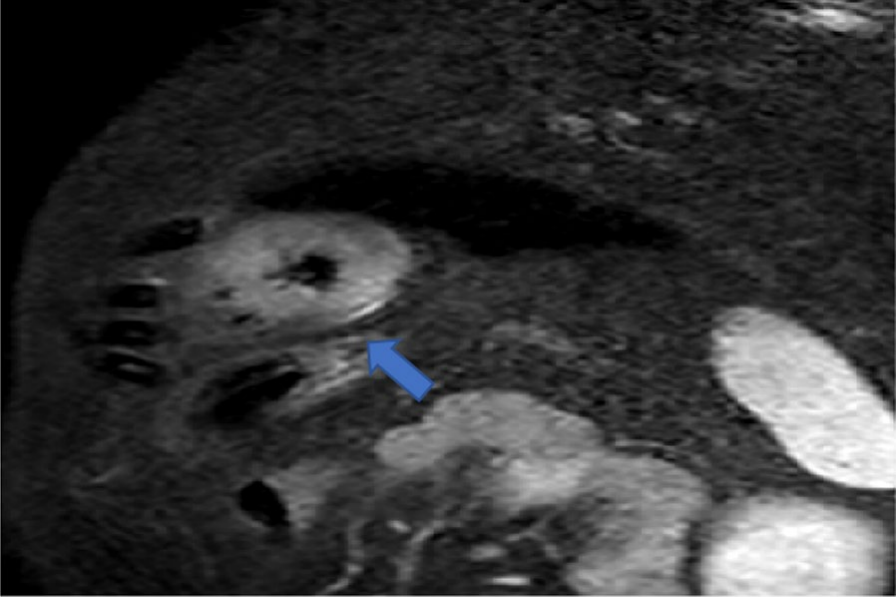
CMR, T2 weighted image in short axis view at the level of the papillary muscles, showing areas of regional increase in the signal intensity (myocardium and the covering pericardium) (arrow), indicative of edema and acute inflammation, from one of our cases

For edema detection, localized areas of myocardial T2 hyperintensity were sought for and reported when seen in 2 perpendicular views. When no localized myocardial T2 hyperintensity is visible, the increase in global T2 signal was searched for by an increased ratio of signal intensity in the myocardium relative to a reference region in skeletal muscle within the same image (with a ratio of ≥ 2.0 considered abnormal) [17] (Fig. 2).

**Fig. 3:**
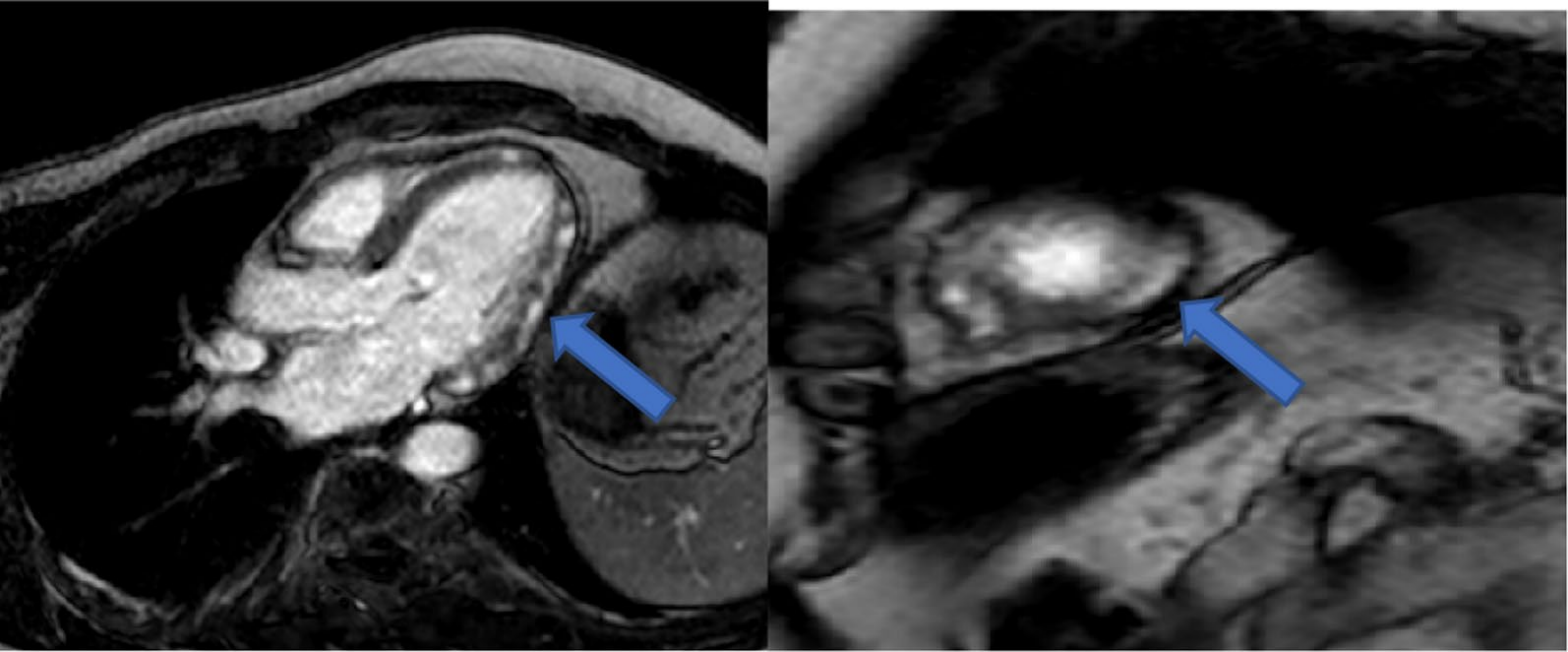
CMR, T1 based phase sensitive inversion recovery images, in vertical long axis view and short axis view for the same patient in Fig. 1, 10–20 min after 0.2 mg/kg gadolinium-based dye intravenous injection, showing mid myocardial–sub-pericardial patches of enhancement (arrows), a pattern typical for myocarditis

For LGE images non-ischemic patterns, typically, sub pericardial and/or mid-myocardial patches, were included. Other patterns such as the ischemic pattern were also reported (Fig. 3).

#### Laboratory investigation

Complete blood count (hemoglobin level, white blood cell count, including differential count to detect eosinophilia), troponin I level, renal chemistry, liver function test, prothrombin time, and concentration, international normalized ratio, and electrolyte levels were all determined for all candidate patients.

#### EMB

EMB was obtained from 12 patients only if they are positive for myocarditis on CMR examination. EMB was performed using a 6-Fr femoral artery sheath; then, a JR3.5 guiding catheter was inserted into the left ventricle. Coke® bioptomes were used to take at least three biopsy samples from the left ventricle from the interventricular septum, apex, and lateral wall under fluoroscopic guidance in the right anterior oblique and left anterior oblique views [18, 19]. Moreover, 2500 IU of heparin was administered in the sheath at the beginning of coronary angiography in all patients. At the end of EMB, a mandatory 2D echocardiography assessment was conducted for the exclusion of any complications, including pericardial effusion or new-onset mitral incompetence due to chordal involvement. Biopsy samples were used for PCR assessment and pathological confirmation of myocarditis in all samples.

#### Serological test

This was conducted at the Microbiology and Immunology Department laboratory at a University hospital.

##### Virus serology

One serum sample for virus serology was collected from each patient. Follow-up serum samples were collected after the initial serum sample. Acute viral infection was diagnosed by serological detection of IgM in the initial sample using ELISA. ELISA kits for the detection of parvovirus B19 (PVB19), coxsackie B virus (CV), and Epstein-Barr virus (EBV) antibodies were purchased from SERION ELISA classic (Germany), and anti-human herpesvirus 6 (HHV-6) were detected using HHV-6 ELISA Kit (Glory, USA). All assays were conducted according to the manufacturer’s instructions.

##### Detection of viral genomes by PCR

Viral nucleic acid was extracted from serum samples using the mini-elute viral nucleic acid extraction kit (Qiagen, Germany). PCR was used for the detection of parvovirus B19, CV, EBV, and HHV-6. Since coxsackieviruses are RNA viruses, reverse transcriptase-PCR (RT-PCR) was used to evaluate these viruses. First-strand cDNA was generated from extracted total viral nucleic acid using a high-capacity cDNA synthesis kit (Applied Biosystems, USA) in a total volume of 20 μL. Three microliters of this first-strand cDNA were combined with 25 pmol/L of each primer and 2X Red Mix Taq Mastermix (Bioline, UK). Forty rounds of amplification were performed under the following conditions: 95 °C for 30 s, 57 °C for 30 s, and 72 °C for 1 min using Veriti Thermocycler (Applied Biosystems, USA). For other DNA viruses, 3 μL of extracted viral nucleic acid was analyzed under the same conditions as the previous. For HHV-6, the conditions were 95 °C for 1 min, 55 °C for 1 min, and 72 °C for 1 min.

#### Clinical follow-up

All patients were followed up for 6 months post-discharge. Full history and clinical examination with detailed echocardiography assessment were done. Major adverse cardiac events were reported including death, rehospitalization for heart failure, and cerebrovascular accidents. Echocardiography results were reported mainly concentrating on the improvement in LV function by an independent operator blinded to the patient's previous clinical status.

### Statistical analysis

Data were verified, coded by the researcher, and analyzed using IBM-SPSS 24.0 (IBM-SPSS Inc., Chicago, IL, USA)^*^. Descriptive statistics: means, standard deviations, medians, ranges, frequency, and percentages were calculated. Test of significances: chi-square test, Fisher’s exact test, and Monte Carlo exact test was used to compare the difference in the distribution of frequencies among different groups. Mann–Whitney U test was calculated to test the differences in the median of continuous variables between groups. Multivariate logistic regression analysis was used for the detection of factors influencing the complete recovery of LVEF at 6 months follow-up. A *P* value < 0.05 was considered significant.

## Results

The flowchart of the study processing is presented in Fig. 1.

### Patient characteristics

The baseline demographic and clinical characteristics of the study group are summarized in Table 1. A total of 2163 patients were admitted to our hospital within 6 months. Fifty-one patients (2.4%) who had suspected myocarditis based on the ESC criteria, were finally included in our study. Males represented 72.5% of patients, with a mean age of 39 ± 16 years. Geographically, all patients were from upper Egypt, most of them (68%) were from rural areas. Regarding the clinical presentation, most patients presented with dyspnea grade II to III for a duration varying from 2 to 8 weeks (40%) and sinus tachycardia (87%).

**Table 1:** Demographic and clinical characteristics of the study group

Variable	Category	Group A (n = 12/47)	Group B (n = 35/47)	*P* value
Age in years	Median (IQR)	38.5 (27)	36 (23)	= 0.456
Sex	Male	8/4	25/10	= 0.511
DM	Yes	1 (8.3%)	3 (8.6%)	= 0.743
HTN	Yes	0 (0%)	3 (8.6%)	= 0.404
Rural	Yes	7 (14.9%)	26 (55.3%)	0.03
Urban	Yes	5 (10.6%)	9 (19.1%)	
SBP (mmHg)	Median (IQR)	120 (18)	110 (40)	= 0.110
DBP (mmHg)	Median (IQR)	80 (8)	70 (20)	= 0.029
Time from start of symptoms to sample collection/days	Median (IQR)	3 (12)	21 (20)	< 0.001
Time from start of symptoms to MRI/days	Median (IQR)	3 (12)	21 (20)	< 0.001
Presenting symptoms	Dyspnea	2 (16.7%)	17 (48.6%)	= 0.042
	Angina	9 (75%)	5 (14.3%)	< 0.001
	Pulmonary edema	0 (0%)	4 (11.4%)	= 0.294
	Palpitation	2 (16.7%)	3 (8.5%)	= 0.583
	Lower limb edema	0 (0%)	2 (5.7%)	= 0.550
	Shock	0 (0%)	9 (25.7%)	= 0.166
ECG finding	Sinus tachycardia	10 (21.3%)	31 (66%)	= 0.428
	ST segment raising	7 (14.9%)	6 (12.8%)	0.550
	Atrial fibrillation	0 (0%)	2 (4.2%)	0.550
	Ventricular tachycardia	2 (16.6%)	1 (2.8%)	= 0.798
	Left bundle branch block	0 (0%)	6 (17.1%)	= 0.044
	Right bundle branch block	0 (0%)	1 (2.8%)	= 0.852
EF% at presentation	Median (IQR)	55% (19)	40% (19)	= 0.007
	≤ 40%	2 (16.7%)	13 (37.1%)	= 0.019
	41–49%	2 (16.7%)	14 (40%)	
	≥ 50%	8 (66.6%)	8 (22.9%)	
ESC position statement criteria	Median	5	4	0.1
Previous COVID by (history)	N (%)	1(2%)	9 (25.7%)	= 0.027
No previous COVID (by history)		11	26 (74.3%)	
Troponin	Positive	11 (91.7%)	25 (71.4%)	= 0.150
Treatment given to the Patients				
	Digoxin/digitoxin	0 (0%)	1 (2.9%)	= 0.745
	ACE inhibitor /angiotensin receptor blocker	5 (41.7%)	19 (54.3%)	= 0.450
	Beta blockers	5 (41.7%)	18 (51.4%)	= 0.450
	Diuretics	3 (25%)	23 (65.7%)	= 0.020
	Spironolactone	3 (25%)	19 (54.3%)	= 0.079
	Amiodarone	2 (16.7%)	3 (8.6%)	= 0.590
	Steroids	4 (33.3%)	3 (8.6%)	= 0.038
	NSAIDs	6 (50%)	5 (14.3%)	= 0.020
	Colchicine	6 (50%)	5 (14.3%)	= 0.020

*We classified patients into two categories based on CMR results*: group A (CMR-positive myocarditis, median score = 5), 12 patients (25.5%), and group B (CMR-negative myocarditis, median score = 4), 35 patients (74.5%), those were suspected clinically but could not be proven by CMR. Four patients were proven to be ischemic by CMR and thus were excluded. Thereby, the number of patients presenting with clinically suspected myocarditis within the recruitment period was 47 cases (representing 2.2% from total admissions). A major difference between groups A and B, was the delayed presentation of group B, with the mean number of days from symptom onset to presentation being 21 days as compared to 3 days in group A. Thereby, sample collection and CMR study were done at different time intervals from the start of symptoms (Table 1).

### Echocardiography findings

Echocardiography findings are presented in Table 2. Fifteen patients (32%) had mildly reduced ejection fraction (EF) of 40–49%, and 16 patients (34%) had good EF > 50%. Twenty-seven (27) patients (57%) had global hypokinesia. Pericardial effusion was found in seven patients (15%).

**Table 2:** Echocardiographic characteristics of the study group

Variable	Category	n = 51	Group A (n = 12 /47)	Group B (n = 35 /47)	*P* value
Ejection fraction	< 40%	19 (37.3%)	2 (16.7%)	13 (37.1%)	
	40–49%	16 (31.4%)	2 (16.7%)	14 (40%)	= 0.019
	> 50%	16 (31.4%)	8 (66.6%)	8 (22.9%)	
End diastolic diameter, cm	Median (IQR)	5.9 (1.5)	5 (0.6)	6 (1.2)	= 0.013
End systolic diameter, cm	Median (IQR)	4.5 (1.6)	3.8 (0.8)	4.8 (1.2)	= 0.005
Left atrial size, cm	Median (IQR)	4 (1)	3.5 (0.5)	4.1 (0.6)	= 0.014
Fraction shortening, %	Median (IQR)	21 (10)	27.5 (14)	19 (10)	= 0.013
Stroke Volume Index	Median (IQR)	40 (11.5)	43 (14)	40 (11)	= 0.077
Cardiac Index	Median (IQR)	3.3 (0.8)	3.4 (0.8)	3.3 (0.9)	= 0.751
Segmental wall motion abnormalities	Yes	9 (17.6%)	5 (41.7%)	4 (11.4%)	
	No	11 (21.6%)	5 (41.7%)	6 (17.2%)	= 0.001
	Global hypokinesia	31 (60.8%)	2 (16.6%)	25 (71.4%)	
Pericardial effusion	Yes	7 (13.7%)	1 (8.3%)	6 (17.1%)	= 0.417

### Microbiological results

More than half of the cases (n = 31/47, 66%) were associated with antibodies against the common cardiotropic viruses: parvovirus B19 IgM 22 (47%), coxsackievirus IgM 16 (34%), and HHV-6 IgM 1 (2%). No evidence for EBV or CMV was found. Eight cases (17%) showed multiple infections. Serum PCR yielded the presence of PVB19 DNA in 16 / 47 (34%) and coxsackievirus DNA in 7 / 47 (15%) of the sample cases.

### Short-term follow-up and predictors of outcome

At 6 months of clinical follow-up of our patients, 91.5% of group A and 26% of group B had recovered left ventricular EF. Only three patients died on follow-up, and all were from group B (Table 3).

**Table 3:** 6 months clinical follow-up of the study group

Clinical follow-up	Group A (n = 12)	Group B (n = 35)	*P* value
Recovered ejection fraction	11 (91.5%)	9 (25.7%)	
Unchanged ejection fraction	0 (0%)	18 (74.3)	= 0.036
Neurological residual	0 (0%)	2 (5.7%)	
Poor ejection fraction	1 (8.5%)	12 (37.1%)	
Died	0 (0%)	3 (8.5%)	

Univariate logistic regression analysis included all available risk factors affecting the improvement of LVEF; identified that the presence of localized SWMA rather than global left ventricular hypokinesia (OR = 9.7; 95%CI = 2.1–44.2; *P* = 0.003), signs of myocarditis detected on CMR (OR = 4.7;95%CI = 1.9–24.7; *P* = 0.04) and a smaller left atrial area (OR = 0.277; 95% CI = 0.0072–0.963; *P* = 0.047) were independent predictors of complete LVEF recovery at 6 months follow-up. (Table 4).

**Table 4:** Independent predictors of complete EF recovery: univariate logistic regression model

Predictor	OR (95% CI)	*P* value
Presence of localized SWMA	9.7 (2.1–44.2)	= 0.003
Myocarditis by CMR	4.7 (1.9–24.7)	= 0.04
Decreasing LA size/cm	0.277 (0.072–0.963)	= 0.047
Increasing ESC PSC	0.51 (0.25–1.03)	= 0.06
Residence (Rural)	3.6 (0.86–15.5)	= 0.07
COVID Status (Positive)	0.46 (0.1–2.06)	= 0.31
Age/years	0.963 (0.914–1.015)	= 0.22
Sex (Male)	1.3 (0.36–4.8)	= 0.66
Seropositive	0.993 (0.221–2.459)	= 0.469
DM	1.5 (0.19–11.9)	= 0.68
HTN	0.72 (0.06–8.5)	0.79

Univariate logistic regression analysis included all available risk factors affecting the improvement of LV EF; identified that the presence of localized SWMA rather than global left ventricular hypokinesia (OR = 9.7; 95%CI = 2.1–44.2; *P* = 0.003), signs of myocarditis detected on CMR (OR = 4.7;95%CI = 1.9–24.7; *P* = 0.04) and a smaller left atrial area (OR = 0.277; 95% CI = 0.0072–0.963; *P* = 0.047) were independent predictors of complete LVEF recovery at 6 m. follow-up

OR, odds ratio; CI, confidence interval; CMR, cardiac magnetic resonance imaging; ESC PSC, European society of cardiology position statement criteria SWMA, segmental wall motion abnormalities; LA; left atrium; DM, diabetes miletus; HTN: hypertension

## Discussion

The main findings of our study are:

- The percentage of patients admitted to our hospital with clinically suspected myocarditis in the period of recruitment was 2.2% among all admissions.
- Out of those, 25.5% had CMR-positive myocarditis and 74.5% had CMR-negative myocarditis.
- Parvovirus B 19 and coxsackievirus were the most endemic viruses that cause viral myocarditis in our society in the current era.
- Moreover, 91.5% of patients with CMR-positive myocarditis recovered their left ventricular function at 6 months follow-up.

Regarding the epidemiological criteria of our population, the mean age was 39 ± 16 with 72.5% males, which is consistent with other recent global reports [20] and 68% were from rural rather than urban areas. Regarding the clinical presentation, significant differences existed between groups A and B. Most of group A (75%) presented with angina-like symptoms vs only (14%) of group B. While heart failure presentation was found in 65.7% of group B (including dyspnea, pulmonary edema, and lower limb edema) vs only 16.7% of group A. Group A patients sought medical advice earlier than group B, (3 vs 21 days), which could be largely explained by the difference in the acuity of the presenting symptoms. (Table 1).

In previous work from our center, we examined the percentage of myocarditis using (unexplained cardiomyopathy) as an inclusion criterion. Based on that, we defined 15 out of 1100 patients (1.4%) as suspected of myocarditis [21]. The different inclusion criteria could largely explain the difference. Other explanations may include: First, our center has largely expanded since then and gradually became a referral center for up to 30 million inhabitants in upper Egypt.

Second, given the lack of a confirmatory investigation for the COVID-19 status and although most of our patient population (79%) denied a previous COVID-19 infection, it is difficult to take this for granted under the current pandemic circumstances. Many studies suggest that subclinical COVID-19 infections are occurring more frequently than expected [22]. One meta-analysis revealed that the pooled prevalence of myocardial injury during the COVID-19 pandemic was 22.33% [23]. Thus, this increase could still be partially explained by the COVID-19 pandemic.

In the current study, we introduced the use of cardiac MRI in all patients. CMR was not available at our center back in 2014. CMR has an important role in patients presenting with unexplained HF and those with an infarct-like presentation with non-obstructive CAD. Its ability of tissue characterization can help in revealing the underlying etiology and sometimes offer some prognostic information [24, 25].

In our study, CMR revealed an ischemic etiology in four patients (8% of the total of 51 patients), based on the characteristic LGE distribution pattern of subendocardial enhancement of variable degrees, along one or more specific coronary distribution [26]. Of the remaining 47 patients, 25.5% showed evidence suggestive of myocarditis.

However, the diagnostic accuracy of CMR in the diagnosis of myocarditis varies widely among reports, depending on many variables. Some important factors are the timing of imaging after symptom onset; clinical presentation; the mechanism and extent of myocardial cell necrosis, if any. In our population, the presentation of group B was later than that of group A, thereby, the CMR study was done significantly later (3 vs 21 days for groups A and B respectively, *P* value < 0.001). Also, CMR diagnostic sensitivity was found to be high for infarct-like presentations, which represented 75% of group A, low for cardiomyopathy-like presentations which were more in group B (Table 1), and very low for arrhythmia presentations [27, 28].

Another important factor is the protocol used. The addition of parametric CMR modalities, such as T1 and T2 mapping and strain modalities, further increased the sensitivity of detection of subtle abnormalities and facilitated the objective assessment of myocardial inflammation or diffuse myocardial fibrosis. However, due to the lack of availability in many centers, current recommendations are still based on the assessment of the basic  CMR parameters [17]. Still, even with the use of these techniques in routine clinical practice, many patients are found to have borderline ‘normal’ T1 and T2 mapping values, which potentially leads to the false-negative exclusion of myocarditis [27]. These facts can explain the negative CMR findings in group B, despite fulfilling the ESC clinical and other lab and/or imaging criteria, with the absence of an obvious alternative diagnosis.

Regarding our serology findings, 31 patients (66%) were found to have immunoglobulins positive for the tested viruses. Most positive cases were noted between parvovirus B19 (47%), the most prevalent in our population, and coxsackievirus (34%), a member of the enteroviruses.

As previously mentioned, historically, virus-induced myocarditis was mostly associated with enterovirus and adenovirus infections. Gradually, this pattern shifted predominantly toward parvovirus B19 and HHV-6 infection [29, 30]. However, this is still highly variable and particularly varies according to the geographic location and temporal differences, as other recent studies still showed a higher prevalence of enteroviruses, particularly, Coxsackievirus in some regions [5, 13, 16].

Our results suggest that enteroviruses as a cause of myocarditis are still highly prevalent in our society, coming second to Parvovirus, which is the most prevalent type, however, larger studies in multiple centers in Egypt are required to confirm this finding.

Four patients were found to have positive CMR features, with negative serology for the tested cardiotropic viruses. Explanations could include viral causes other than those tested particularly the coronavirus, non-viral and noninfectious causes, or chronic inflammatory cardiac affection, where IgM of specific viruses are no longer detected.

Given the challenging diagnosis of myocarditis, efforts are continuing to introduce new simple techniques that might help in the diagnosis in everyday practice, where the EMB as a gold standard is not always available. An example is the recently suggested ECG feature, namely QRS fragmentation, as a possible electrocardiographic diagnostic marker in patients with acute myocarditis [31, 32].

On follow-up of our population, the majority of group A recovered normal EF by echocardiography at 6 months, further confirming the diagnosis. The three patients who died were from group B.

Our results showed that LGE-positive cases on CMR had a good outcome at 6 months. That was not the case in many other studies. Recent studies showed that the prognostic role of CMR is related to the presence of LGE, its location, extent, pattern, and distribution, together with the LV volumes and EF among other factors, with mid-wall septal LGE having a particular role [5].

Most of these studies addressed long-term outcomes up to 10 years, which is not the case with our study. Also, the small number of patients in our study, the fact that most of them did not have marked LV dilatation at baseline as an inclusion criterion, which was more evident in group A, and that they were hemodynamically stable at presentation and all over the hospitalization course, all these factors could explain the discrepancy.

We also found that patients with a previous history of COVID-19 infection showed a worse prognosis. The issue of myocarditis in COVID -19 patients is still debatable, regarding the mechanism, diagnosis, and outcome [33]. Besides, none of our patients were in a clinically active COVID infection at the time of evaluation. This further complicates the comparison to other studies addressing the topic of COVID-related myocarditis. Further studies directly addressing this subject, are needed.

There are many terms used for the description of myocarditis, based on clinical presentation, time, etiology, and pathophysiology [34].

The differences between groups A and B in clinical presentation, echocardiography and CMR findings, and prognosis, come in agreement with the different definitions of acute myocarditis and chronic inflammatory cardiomyopathy, while both still fulfill the ESC position statement criteria. Chronic inflammatory cardiomyopathy may represent the evolution of ≥ 1 acute myocarditis episode that, either diagnosed or missed in the acute phase, caused myocardial damage and systolic dysfunction [34].

Given the wide spectrum of causative agents, clinical manifestations, and geographic differences of such a condition, there are recommendations to adopt an international, cooperative, and consistent approach to characterize myocarditis and differentiate its different types and stages. This global approach should be comprehensive and as standardized as possible from a virologic, immunological, pathological, and clinical view [29].

*There are some limitations of our study*, the small number of patients from a single center, the lack of specific testing for COVID-19 status, the lack of parametric imaging on CMR, the fact that we did the EMB only for those with CMR positive cases, not the whole population, and the lack of long term follow up.

## Conclusion

The percentage of patients admitted to our hospital with clinically suspected myocarditis in the recruitment period was 2.2%. CMR is only a good positive test for the diagnosis of acute myocarditis. Since the EMB as a gold standard technique for diagnosis of myocarditis, is not always feasible, a combination of clinical, ECG, laboratory, and imaging features is important in the diagnostic workup of such a challenging condition.. Parvovirus B19 and coxsackievirus were the most common pathogens in our locality. (Clinical trial registration no., NCT04312490; STDF grant no., 26393).

## Data Availability

The datasets used and/or analyzed during the current study are available from the corresponding author on reasonable request.
